# Self-Limiting Layer Synthesis of Transition Metal Dichalcogenides

**DOI:** 10.1038/srep18754

**Published:** 2016-01-04

**Authors:** Youngjun Kim, Jeong-Gyu Song, Yong Ju Park, Gyeong Hee Ryu, Su Jeong Lee, Jin Sung Kim, Pyo Jin Jeon, Chang Wan Lee, Whang Je Woo, Taejin Choi, Hanearl Jung, Han-Bo-Ram Lee, Jae-Min Myoung, Seongil Im, Zonghoon Lee, Jong-Hyun Ahn, Jusang Park, Hyungjun Kim

**Affiliations:** 1 School of Electrical and Electronic Engineering, Yonsei University, Seoul 120-749, Korea; 2 School of Materials Science and Engineering, Ulsan National Institute of Science and Technology (UNIST), Ulsan 689-798, Korea; 3 Department of Materials Science and Engineering, Yonsei University, Seoul 120-749, Korea; 4 Institute of Physics and Applied Physics, Yonsei University, Seoul 120-749, Korea; 5 Department of Materials Science and Engineering, Incheon National University, Incheon 406-772, Korea

## Abstract

This work reports the self-limiting synthesis of an atomically thin, two dimensional transition metal dichalcogenides (2D TMDCs) in the form of MoS_2_. The layer controllability and large area uniformity essential for electronic and optical device applications is achieved through atomic layer deposition in what is named self-limiting layer synthesis (SLS); a process in which the number of layers is determined by temperature rather than process cycles due to the chemically inactive nature of 2D MoS_2_. Through spectroscopic and microscopic investigation it is demonstrated that SLS is capable of producing MoS_2_ with a wafer-scale (~10 cm) layer-number uniformity of more than 90%, which when used as the active layer in a top-gated field-effect transistor, produces an on/off ratio as high as 10^8^. This process is also shown to be applicable to WSe_2_, with a PN diode fabricated from a MoS_2_/WSe_2_ heterostructure exhibiting gate-tunable rectifying characteristics.

Two-dimensional (2D) materials, and the heterostructures that can be created from them, have been widely studied due to their atomic-scale thickness, flexibility and unique electrical/optical properties^1^,^2^,^3^,^4^,^5^. However, there is still a need to develop a layer-controlled synthesis method capable of producing a uniform 2D material over large areas in order to ensure the reliable operation of optoelectronic devices whose properties are dependent on the number of 2D material layers. The well-established CVD process has allowed large-area graphene sheets to be used in various practical applications^6^,^7^, as this process is self-limited through a surface-catalyzed process based on the lower solubility of carbon in Cu than in Ni^8^. Since it is this self-limiting behavior that makes it possible to achieve monolayer (1L) graphene over 95% of the target growth area^8^, achieving a similar self-limiting behavior is clearly an important first step in the development of any new process for the large-area uniform growth of 2D materials.

Transition metal dichalcogenides (TMDCs) and their relevant 2D heterostructures (e.g., MoS_2_/WSe_2_ and MoS_2_/graphene) have been the most heavily studied semiconducting 2D materials^3^,^4^,^9^,^10^,^11^,^12^,^13^. Most recent research has been devoted to synthesizing uniform and layer-controlled TMDCs over large areas^9^,^14^,^15^, such as chemical vapor deposition and transformation of Mo and MoOx thin film^16^,^17^,^18^, but unlike graphene, the self-limiting growth of TMDCs with wafer-level layer controllability and uniformity has not yet been achieved. Atomic layer deposition (ALD) is known to be self-limiting, as the growth rate is dependent on the adsorption of precursor molecules rather than growth conditions such as exposure time^19^,^20^, but as growth occurs through the formation of multi-layer islands it is difficult to achieve the layer controllability needed when compared to other techniques such as CVD^21^,^22^. Maximizing the self-limiting behavior of the ALD process is therefore essential to achieving the layer controllability needed for a 2D structure, which requires not only careful optimization of the process conditions (e.g., temperature, pressure, exposure of precursor/reactant), but also the careful selection of the precursor and reactant^23^,^24^. Moreover, since the ALD process is entirely based on surface reaction, it is important to understand the surface characteristics of the material being deposited. For example, the ALD of metal oxides or metals on graphene is made difficult by the chemically inactive nature of the graphene surface^25^,^26^,^27^. As 2D TMDCs also have a chemically inactive surface, it is reasonable to expect they will exhibit a unique growth behavior during ALD when compared to conventional materials that are rich in dangling bonds^28^.

In this study, the self-limiting layer synthesis (SLS) of a 2D TMDC (MoS_2_) is achieved through ALD by combining precursor exposure, purging, reactant exposure and a final purging into a single cycle. In this way, a point is reached at which the number of layers produced is determined purely by the growth temperature; a unique behavior that is directly attributable to the chemical inactivity of the 2D MoS_2_ surface. The characteristics and layer uniformity achieved are subsequently assessed through spectroscopic and microscopic analysis, and the universality of the process itself is tested by applying it to the fabrication of a MoS_2_/WSe_2_ heterostructure for use in a diode.

## Self-limiting layer synthesis of MoS_2_

**Figure 1:**
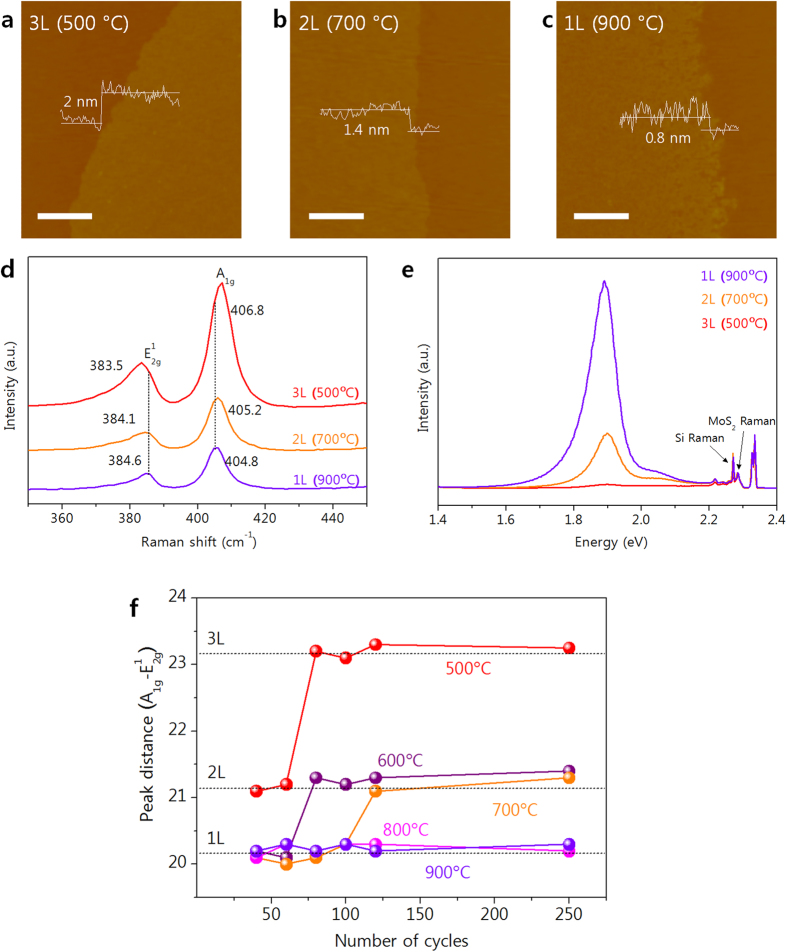
AFM images and height profiles of (**a**) tri-, (**b**) bi- and (**c**) mono-layers of MoS_2_ transferred onto a SiO_2_ substrate (scale bar = 0.5 μm).(**d**) Raman spectra and (**e**) PL spectra of tri-, bi- and mono-layer MoS_2_ on SiO_2_. (**f**) Raman peak distances for MoS_2_ with various SLS cycles and growth temperatures.

Figure 1(a–c) contains AFM images and height profiles of MoS_2_ synthesized through 120 ALD cycles at growth temperatures of 500, 700 or 900 °C. By transferring this MoS_2_ to new SiO_2_ substrates it was found that the thickness produced was 2 nm at 500 °C, 1.4 nm at 700 °C and 0.8 nm at 900 °C, which corresponds to the thickness of tri-, bi-, and mono-layer (3L, 2L, and 1L) MoS_2_^29^. The Raman spectra (λ_exc_ = 532 nm) in Fig. 1(d) shows that the 1L MoS_2_ exhibits E^1^_2g_ and A_1g_ modes from in-plane and out-of-plane vibrations at 384.6 cm^−1^ and 404.8 cm^−1^, but these shift to 384.1 and 405.2 cm^−1^ with 2L MoS_2_, and to 383.5 and 406.8 cm^−1^ with 3L MoS_2_. The peak distance between E^1^_2g_ and A_1g_ is often used to determine the number of MoS_2_ layers, as an increase in layers is accompanied by a softening of the E^1^_2g_ mode frequency and a stiffening of the A_1g_ mode frequency^30^,^31^. In this case, the calculated peak distances of 20.2 cm^−1^ for 1L, 21.1 cm^−1^ for 2L, and 23.3 cm^−1^ for 3L all agree well with previously reported values for MoS_2_^29^,^32^,^33^. Thus, both the AFM and Raman results show that it is the growth temperature that determines the number of MoS_2_ layers by SLS.

The PL spectra of the synthesized MoS_2_ are shown in Fig. 1(e) as a function of the number of layers obtained. Note that the 1L MoS_2_ spectrum exhibits a strong PL signal at 1.89 eV and a weak, wide PL signal at 2.05 eV, which correspond to the A_1_ and B_1_ direct excitonic transitions of MoS_2_^14^,^34^. These signals weaken in the case of 2L MoS_2_, and become negligible with 3L MoS_2_, as the increasing number of layers induces a transition from a direct to an indirect band gap. This is concordant with previous results regarding the dependence of the PL signal on the number of layers^14^,^34^,^35^ and further confirms the growth temperature dependent nature of the SLS of MoS_2_.

Given the good correlation between the Raman peak distances and the number of layers of MoS_2_ obtained on a SiO_2_ substrate, this was used a criterion to assess the effects of varying the number of process cycles from 40 to 250 at growth temperatures of 500, 600, 700, 800 and 900 °C (the Raman spectra for each point are presented in Supplementary Fig S2). From the results shown in Fig. 1(f), it is evident that the number of MoS_2_ layers does not increase linearly with the number of process cycles, but rather saturates at a certain critical point determined by the synthesis temperature (500 °C for 3L, 600–700 °C for 2L, and 800–900 °C for 1L). This stands in stark contrast to conventional ALD, in which the thickness does in fact increase linearly with the number of process cycles. We can therefore only conclude that the growth mechanism of the current SLS process is totally different from that of conventional ALD.

This peculiar “self-limiting” behavior of ALD during the SLS process is believed to be caused by the inherently chemically inactive nature of the surface of TMDCs such as MoS_2_. Specifically, during the growth of the first layer, precursor molecules (MoCl_5_ in this case) chemically adsorb to the abundant adsorption sites on the SiO_2_ surface. However, once this initial layer is formed over the entire surface, any further chemical adsorption of precursor molecules is hindered by the absence of suitable adsorption sites on the newly created TMDC surface^28^,^36^. Synthesis is therefore forced to proceed through the physical adsorption of MoCl_5_ molecules on MoS_2_; with the adsorption/desorption of precursor molecules under this physical adsorption-dominant regime being determined by the growth temperature. This can perhaps be better explained by the framework of the Lennard-Jones potential model: i.e., at lower temperatures molecules are trapped in a potential well because their thermal energy is less than the potential depth, whereas at higher temperatures they have sufficient thermal energy to escape^28^,^36^.

The surface potential of MoS_2_ that is induced by the positive charge between it and the SiO_2_ substrate also affects the potential depth of the precursor molecules; the surface potential of MoS_2_ decreasing with an increasing number of MoS_2_ layers due to their screening effect on the electric field^37^. This decrease in surface potential can reduce the potential depth of the MoCl_5_ molecule on MoS_2_ in the same way that the surface potential of physically adsorbed CH_4_ on h-BN decreases with an increasing number of layers^38^. In other words, once a specific number of MoS_2_ layers has been formed at any given growth temperature, any MoCl_5_ molecules adsorbed onto the MoS_2_ basal plane can be easily desorbed due to the reduced potential depth, thereby creating a self-limiting growth behavior.

It should be noted here that the SLS of 2D MoS_2_ relies on using a sufficiently high process temperature to ensure the formation of layered 2D structure with chemically inactive surface. The lower crystallinity and non-layered 3D structure at lower growth temperatures causes deposition to proceed through chemical adsorption of precursor molecules, as is the case in the conventional ALD of MoS_2_ without self-limiting growth behavior^21^,^22^. These temperature requirements make proper selection of the precursor essential, with MoCl_5_ being used in this study due to its higher thermal stability relative to other metal organic precursors.

**Figure 2:**
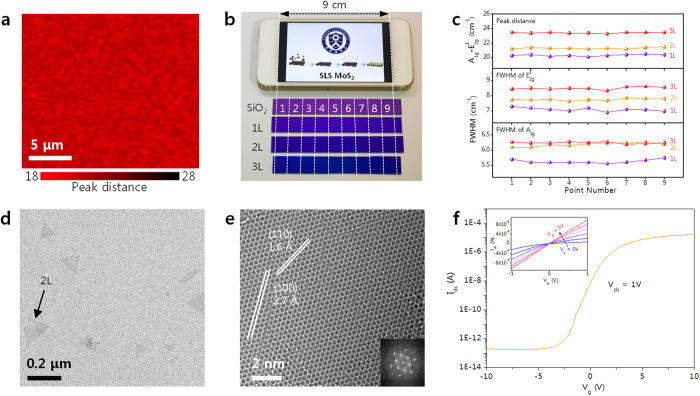
(**a**) Raman map of peak distance between E^1^_2g_ and A_1g_ modes for monolayer MoS_2_ (scale bar = 5 μm). (**b**) Large-area (~9 cm) mono-, bi-, and tri-layer MoS_2_ on a SiO_2_ substrate comparable in size to a cellular phone display screen. (**c**) Relative peak distances and FWHM of E^1^_2g_ and A_1g_ modes for nine measurement points on mono-, bi-, and tri-layer MoS_2_. (**d**) Low-magnification TEM image (scale bar = 0.2 μm) of monolayer MoS_2_ on a TEM grid and (**e**) HRTEM image (scale bar = 2 nm) of the selected region. The inset gives the corresponding FFT pattern. (**f**) Transfer curve and (inset) output curve for a FET fabricated on monolayer MoS_2_.

The uniformity of the MoS_2_ obtained through SLS was evaluated at different scales through Raman mapping of the peak distances between the A_1g_ and E^1^_2g_ modes. The Raman map of the 1L SLS MoS_2_ in Fig. 2(a) shows a perfectly uniform distribution at a micrometer scale (20 μm × 20 μm), with statistical analysis (see Supplementary Fig. S6) revealing the average peak distance and standard deviation to be 20.3 and 0.6, respectively. Uniformity at a wafer-level scale was measured by synthesizing 1L, 2L and 3L MoS_2_ onto 1.5 × 9 cm^2^ SiO_2_ substrates; the substrate size being limited in this instance by the diameter (~3 cm) and length (15 cm hot zone) of the tube furnace used. It is clear from Fig. 2(b) that the color of the SLS MoS_2_ is certainly dependent on the number of layers, but to assess the cm-scale uniformity, Raman spectra were measured at nine different positions along the length of the SLS MoS_2_. The peak distances and full-width at half-maximum (FWHM) of the E^1^_2g_ and A_1g_ modes are plotted in Fig. 2(c) for each position, from which we see that with all samples the variation in peak distance and FWHM of the E^1^_2g_ and A_1g_ modes with position is quite small (~2% for peak distance, ~5 and ~4% for the FWHM of E^1^_2g_ and A_1g_, respectively). In addition, the Raman peak distance varies from 20.2 to 23.4 cm^−1^ as the number of layers is increased, confirming that good uniformity and layer control is achieved at the wafer level through the SLS of MoS_2_.

The crystallinity and electrical performance of the 1L SLS MoS_2_ was evaluated through high-resolution TEM (HRTEM) analysis and by using it in a top-gated field-effect transistor (FET). The low-magnification TEM image of 1L SLS MoS_2_ in Fig. 2(d) reveals triangular, dark-contrast regions of 2L MoS_2_; but as these represent only about 4% of the total area, the 1L SLS MoS_2_ can be considered to have near-perfect ( > 95%) micrometer-scale layer uniformity. This growth of 2L MoS_2_ on 1L MoS_2_ could be due to the energetically favorable adsorption of MoCl_5_ on defect sites such as sulfur vacancies or grain boundaries, as such adsorption of molecules is seen with graphene^26^,^27^. In the HRTEM image of the 1L SLS MoS_2_ in Fig. 2(e), selected regions exhibit a honeycomb-like with a lattice spacing of 0.27 or 0.16 nm depending on whether it involves (100) or (110) planes. A six-fold coordination symmetry is also clearly evident in the fast Fourier transform (FFT) image in the inset of Fig. 2(e). The approximate grain size is 80–100 nm, though this could potentially be improved through further optimization of the process and substrate conditions. The electrical performance of the 1L SLS MoS_2_ was evaluated by using it in the fabrication of a top-gated FET with Au(10 nm)/Ti(50 nm) electrodes and an ALD Al_2_O_3_ (40nm) gate insulator. The room temperature performance of this FET at 10^−5 ^mTorr is shown in Fig. 2(f), which reveals an n-type behavior; the 0.2 cm^2^/V∙s field effect electron mobility in the linear regime of the transfer curve agreeing with a previous report of a MoS_2_ FET^39^. Interestingly, this 1L SLS MoS_2_ FET also has a low subthreshold swing value of ~0.36 V/dec and an excellent on/off current ratio of ~10^8^ that is higher than anything previously achieved with 1L MoS_2_^18^, and is in fact comparable with a single crystal^39^.

### Vertically stacked heterostructure

**Figure 3:**
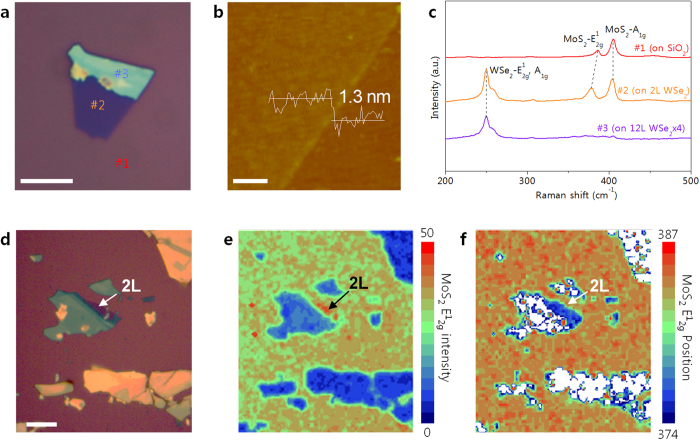
(**a**) OM image of an exfoliated WSe_2_ flake on SiO_2_ (scale bar = 10 μm). (**b**) AFM image and height profile of SLS MoS_2_ on 2L WSe_2 _region (scale bar = 0.5 μm). (**c**) Raman spectra for the numbered regions in Figure 3(a): #1: MoS_2_ on SiO_2_, #2: MoS_2_ on 2L WSe_2_ and #3L SLS MoS_2_ on 12L WSe_2_. (**d**) OM image of SLS MoS_2_ on exfoliated WSe_2_ flakes (scale bar = 10 μm) and corresponding Raman mapping results for (**e**) MoS_2_ E^1^_2g_ intensity and (**f**) MoS_2_ E^1^_2g_ position.

If the proposed self-limiting growth mechanism of SLS is valid, then it would be expected to apply to other 2D materials. This was therefore tested using mechanically exfoliated WSe_2_ flakes on a SiO_2_(300 nm)/Si substrate, as demonstrated by the microscopy (OM) images in Fig. 3(a). This WSe_2_ flake was confirmed through AFM and Raman analysis (See Supplementary Fig. S8(a–c)) to contain regions of both 2L WSe_2_ (#2) and 12L WSe_2_ (#3). The AFM image of the SLS MoS_2_ produced on this WSe_2_ flake at 800 °C (Fig. 3(b)) shows that a thickness of 1.3 nm (or 2L WSe_2_) was obtained, indicating that 1L MoS_2_ is deposited on both 2L WSe_2_ and the SiO_2_ substrate under these conditions. Figure 3(c) shows the Raman spectra obtained at 3 different points of the SLS MoS_2_ on WSe_2_/SiO_2_. In the SiO_2_ region (#1), E^1^_2g_ and A_1g_ Raman peaks for MoS_2_ are observed at 385.2 and 405 cm^−1^, respectively, with the peak distance of 20.2 indicating that 1L MoS_2_ was obtained as expected. Raman peaks of WSe_2_ (i.e., the sum of the E^1^_2g_ and A_1g_ peaks at 249.8 cm^−1^) are observed in the 2L WSe_2_ region (#2) along with peaks for MoS_2_ (E^1^_2g_ at 378.4 cm^−1^ and A_1g_ at 404.8 cm^−1^), indicating that MoS_2_ was also synthesized on the WSe_2_ flake. Furthermore, the absence of any Raman peaks related to MoSe_2_ (E^1^_2g_ at 286 cm^−1^ and A_1g_ at 244 cm^−1^) or WS_2_ (E^1^_2g_ at 356 cm^−1 ^and A_1g_ at 420 cm^−1^) indicates that there is no significant mixing or alloying between the two 2D materials^15^,^40^. There is, however, a notable 7 cm^−1^ downshift in the E^1^_2g_ peak of MoS_2_ in the 1L MoS_2_/WSe_2_ region relative to the MoS_2_/SiO_2_ region. A similar downshift has been reported in the case of an interlayer-coupled 1L MoS_2_/1L WSe_2_ heterostructure fabricated by transferring individual MoS_2_ and WSe_2_ flakes, with this being attributed to interaction between MoS_2_ and WSe_2_^41^. Meanwhile, the absence of any MoS_2_ Raman peaks in the 12L WSe_2_ (#3) region indicates that there is effectively no growth of MoS_2_ on 12L WSe_2_, further supporting the idea that the self-limiting nature of the SLS process is layer dependent.

For further examination of SLS MoS_2_ on WSe_2_, Raman mapping of the MoS_2_ E^1^_2g_ peak intensity and position was compared against OM images of 1L SLS MoS_2_ grown on WSe_2_ flakes on a SiO_2_ substrate. In Fig. 3(d), regions confirmed by Raman analysis and AFM (See Supplementary Fig. S8(d)) to be 2L WSe_2_ are indicated by white arrows, with the rest being bulk WSe_2_. The Raman map of MoS_2_ E^1^_2g_ intensity in Fig. 3(e) shows that a strong MoS_2_ E^1^_2g_ signal is observed only at 2L WSe_2_ regions, indicating that MoS_2_ was not synthesized on bulk WSe_2_. The Raman map of MoS_2_ E^1^_2g_ position (Fig. 3(f)) further supports the notion that MoS_2_ grows only on 2L WSe_2_, which is accompanied by a downshift relative to the MoS_2_ E^1^_2g_ position on SiO_2_. This confirms the validity of using SLS to produce MoS_2_ on other chemically inert surfaces such as WSe_2_, and indicates that the process has the potential for widespread application.

**Figure 4:**
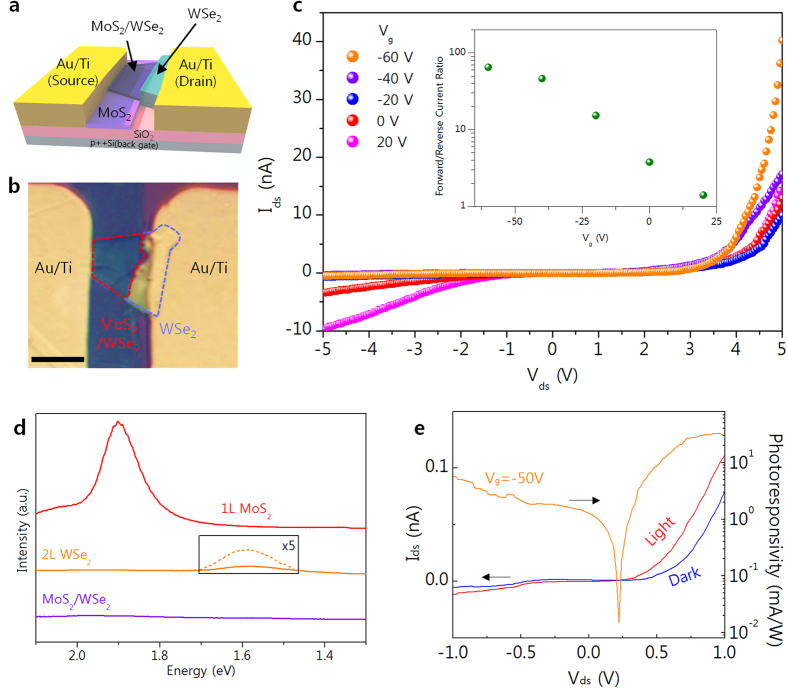
(**a**) Schematic and (**b**) OM image of fabricated PN diode. (**c**) I–V characteristics of PN diode with various gate bias values of between −60 and 20 V, and (inset) forward/reverse current ratio at V_ds_ = |5 V|. (**d**) PL spectra for 1L MoS_2_ (red) 2L WSe_2_ (orange) and a heterostructure created by the two (violet). (**e**) I–V characteristics with (red) and without illumination (blue), and calculated photoresponsivity (orange).

As the SLS process clearly allows for much greater layer control than previously reported methods^10^,^42^,^43^, it represents a promising option for fabricating atomically thin functional devices such as PN diodes, light emitting diodes and inverters^10^,^12^,^44^. To test this, a PN diode was fabricated using a 1L SLS MoS_2_/2L WSe_2_ heterostructure, with Fig. 4(a,b) showing the device structure and an OM image of the fabricated PN diode. Operation of this device is dependent on the back gate voltage, which as shown in Fig. 4(c), can be adjusted by varying the carrier concentration through electrical doping. In other words, the PN diode exhibits a gate-tunable characteristic, with an increase in gate voltage from -60 to 20 V changing the p-n rectifying configuration to n-n junction behavior. The calculated forward/reverse current ratio at V_ds_ = |5 V| clearly shows this gate-tunable PN diode characteristic (inset of Fig. 4(c)). The forward/reverse current ratio of ~80 at V_g_ = -60 V, is higher than previously reported for a PN diode based on 1L MoS_2_/1L WSe_2_ (~50 at V_ds_ = |8|V)^45^, but drops to 1.4 at V_g_ = 20 V. This gate-tunable characteristic could be explained by a variation in carrier density with electrical doping, as consistent with a previous report^45^ (also explain in Supplementary Fig. S10). Also of note is the fact that this PN diode exhibits a strong PL quenching property and photovoltaic effect, indicating a rapid carrier separation at the MoS_2_/WSe_2_ junction^10^,^42^. Figure 4(d) shows the PL spectra for 1L MoS_2_, 2L WSe_2_ and the heterostructure created from them. It is evident from this that the strong PL peak for the direct gap transition of 1L MoS_2_ is greatly suppressed by the WSe_2_ junction, which is attributed to the rapid separation of charge carriers^10^. Figure 4(e) shows the I-V characteristics of a MoS_2_/WSe_2_ PN diode at Vg = -50 V with and without illumination by an incident optical power density of 14 W/m^2^. We see from this that the current increases with illumination due to the generation of optically excited carriers. The open circuit voltage of 0.2 V indicates a photovoltaic effect, with a calculated photoresponsivity of 33 mA/W at V_ds_ = 1 V. As a result, we show the potential of SLS MoS_2_/WSe_2_ structure in photovoltaic device as well as PN diode.

## Conclusion

In summary, the synthesis of MoS_2_ on a SiO_2_ substrate has been successfully achieved through a new self-limiting process that allows the number of layers formed to be controlled by varying the growth temperature. Though the precise mechanism requires further study, this behavior is believed to be caused by the lack of dangling bonds on the surface of MoS_2_ and the screening effect that MoS_2_ layers have on the substrate’s electric field. More importantly, this process can achieve excellent layer uniformity (up to 95%) over large areas at wafer-level scale. The resulting 2D MoS_2_ can produce n-type behavior and a high on/off ratio when used in a top-gated FET, and can be grown on other chemically inert 2D materials such as WSe_2_. Indeed, a PN diode based on a MoS_2_/WSe_2_ heterostructure is capable of a high forward/reverse current ratio, and exhibits a gate-tunable rectifying property attributable to electrical doping by gate voltage. We therefore believe that this new method could be extended to the development of other 2D TMDCs materials and 2D heterostructures.

## Methods

### SLS MoS_2_ Growth

A tube furnace reactor was used to synthesize MoS_2_ directly onto SiO_2_ (285 nm)/Si substrates using MoCl_5_ and H_2_S as the precursor and reactant, respectively. A bubbler containing the precursor was heated to 90 °C to ensure an adequate vapor pressure for the precursor molecules to be carried into the tube by pure argon (99.999%) carrier gas. The SLS cycle consisted of four steps, each with the same with ALD procedure of: precursor exposure for 4 s, a 5s Ar purge, 3 s H_2_S reactant exposure, and a final 5 s Ar purge.

### Transfer of MoS_2_

The as-synthesized MoS_2_ on the SiO_2_ substrate was spin coated with polymethyl methacrylate (PMMA) at 4000 rpm for 60 s. After curing the PMMA at 100 °C for 15 min, the sample was immersed in a 10% HF solution to etch away the SiO_2_ layer. The sample was then washed with DI water and transferred to a new SiO_2_/Si substrate. Finally, the PMMA was removed using acetone and the sample washed with isopropyl alcohol.

### Fabrication of Top-Gated Field-Effect Transistor

The MoS_2_-based FET was fabricated from as-synthesized 1L MoS_2_ on a SiO_2_ (300 nm)/Si substrate by evaporating Au(10 nm)/Ti(50 nm) electrodes and an ALD Al_2_O_3_ (40nm) gate insulator through conventional photolithography and reactive ion (O_2 _plasma) etching.

### Fabrication of PN diode

The 1L SLS MoS_2_ was synthesized on WSe_2_ flakes at 800 °C. Then, PN diode was fabricated from an SLS MoS_2_/WSe_2_ heterostructure on a SiO_2_ (300 nm)/Si substrate by evaporating Au(10 nm)/Ti(50 nm) electrodes. After etching, electrical contacts were formed between the drain electrode and 12L WSe_2_, and between the MoS_2_ and source electrode.

### Characterization of MoS_2_

OM (Nikon ECLIPSE LV100ND), SEM(JEOL-6701F), Raman spectroscopy (HORIBA, Lab Ram ARAMIS; 532 nm laser excitation wavelength), AFM (VEECO, Multimode), PL (SPEX1403, SPEX; 532 nm laser excitation wavelength), XPS (Thermo U.K., K-alpha radiation), TEM (FEI Titan G2 Cube 60-300, accelerating voltage = 80 kV) and a voltage/current meter (Keithley 4200, Keithley Instruments) were all used in characterizing the SLS MoS_2_ nanosheets.

## Additional Information

**How to cite this article**: Kim, Y. *et al.* Self-Limiting Layer Synthesis of Transition Metal Dichalcogenides. *Sci. Rep.* **6**, 18754; doi: 10.1038/srep18754 (2016).

## Supplementary Material

Supplementary Information
