# Supplementary material for: Self-Limiting Layer Synthesis of Transition Metal Dichalcogenides
Source: Sci Rep. 2016 Jan 4;6:18754. doi: 10.1038/srep18754 (PMC4698672; doi:10.1038/srep18754)
Supplement: Supplementary Information [file srep18754-s1.doc]

**Supplementary Information**

Self-Limiting Layer Synthesis of Transition Metal Dichalcogenides

Youngjun Kim,^1†^ Jeong-Gyu Song, ^1†^ Yong Ju Park,^1^ Gyeong Hee Ryu,^2^ Su Jeong Lee,^3^ Jin Sung Kim, ^4^ Pyo Jin Jeon,^4^ Chang Wan Lee,^1^ Whang Je Woo,^1^ Taejin Choi,^1^ Hanearl Jung,^1^ Han-Bo-Ram Lee,^5^ Jae-Min Myoung,^3^ Seongil Im,^4^ Zonghoon Lee,^2^ Jong-Hyun Ahn,^1^ Jusang Park^1^ and Hyungjun Kim^1*^

^1^School of Electrical and Electronic Engineering, Yonsei University, Seoul 120-749, Korea

^2^School of Materials Science and Engineering, Ulsan National Institute of Science and Technology (UNIST), Ulsan 689-798, Korea

^3^Department of Materials Science and Engineering, Yonsei University, Seoul 120-749, Korea

^4^Institute of Physics and Applied Physics, Yonsei University, Seoul 120-749, Korea

^5^Department of Materials Science and Engineering, Incheon National University, Incheon 406-772, Korea

*E-mail: [hyungjun@yonsei.ac.kr](mailto:hyungjun@yonsei.ac.kr)

**A. Optical microscopy and chemical analysis of MoS_2_**

Figure S1(a-c) shows the optical microscopy (OM) images obtained of mono-, bi- and tri-layered SLS MoS_2_. Transferring the MoS_2_ to a new SiO_2_ substrate confirmed it to be very different in color, changing from pale to dark wine as the number of SLS MoS_2_ layers was increased. However, although the as-synthesized MoS_2_ was uniform and continuous, some wrinkles are unavoidably formed during the transfer process.

The XPS spectra at the Mo3d, S2p and Cl2p core levels of the tri-layer MoS_2_ synthesized at 500°C are shown in Figure S1(d). Note that the Mo3d core level has three peaks at 232.5, 229.4 and 226.5 eV, which correspond to the Mo3d_3/2_ and Mo3d_5/2_ levels of the Mo3d and S2s states, respectively. The S2p core level spectrum exhibits two peaks at 163.3 eV and 162.2 eV, which correspond to the S2p_1/2_ doublet and S2p_3/2_, respectively. The Cl2p core level spectrum exhibits no discernible peaks, indicating that the MoS_2_ contained no XPS-detectable Cl species. The calculated stoichiometric ratio is 2 (S/Mo).


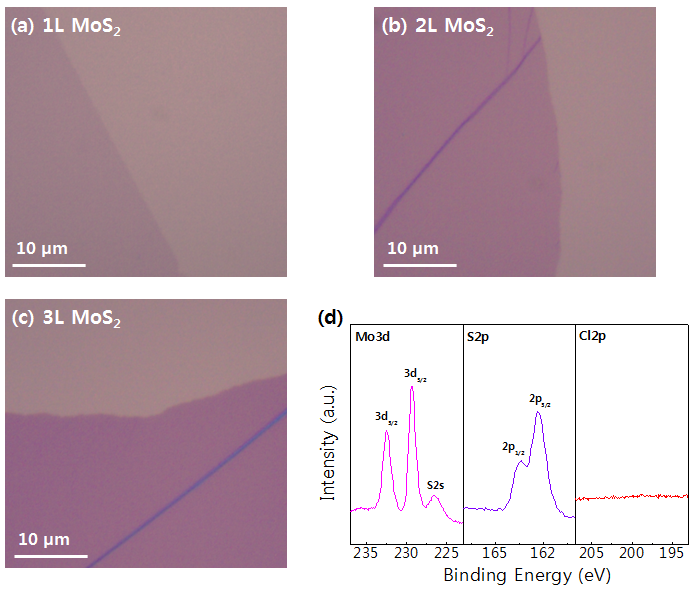


**Figure S1.** Optical microscopy (OM) images of (a) mono-layer (1L), (b) bi-layer (2L), and (c) tri-layer (3L) MoS_2_ after being transferred to the SiO_2_ (285 nm) substrate. (d) XPS measurements for the Mo3d, S2p, and Cl2p core levels of the tri-layer MoS_2_

**B. SEM image of the initial MoS_2_ growth**


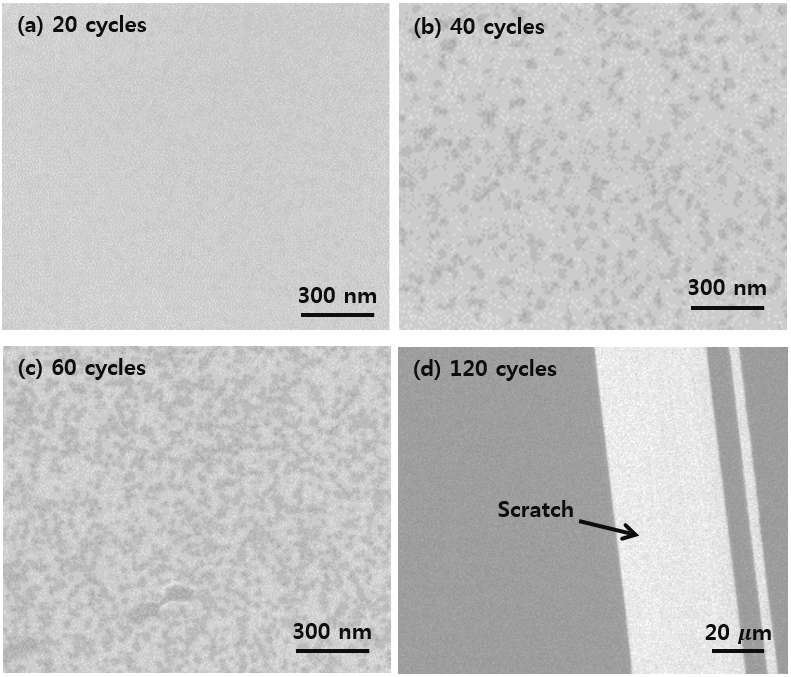


**Figure S2.** SEM images of the obtained MoS_2_ synthesized at 800 ℃ when the ALD cycles are (a) 20, (b) 40, (c) 60, (d) 120 cycles

**C. Effect of increasing the number of process cycles**


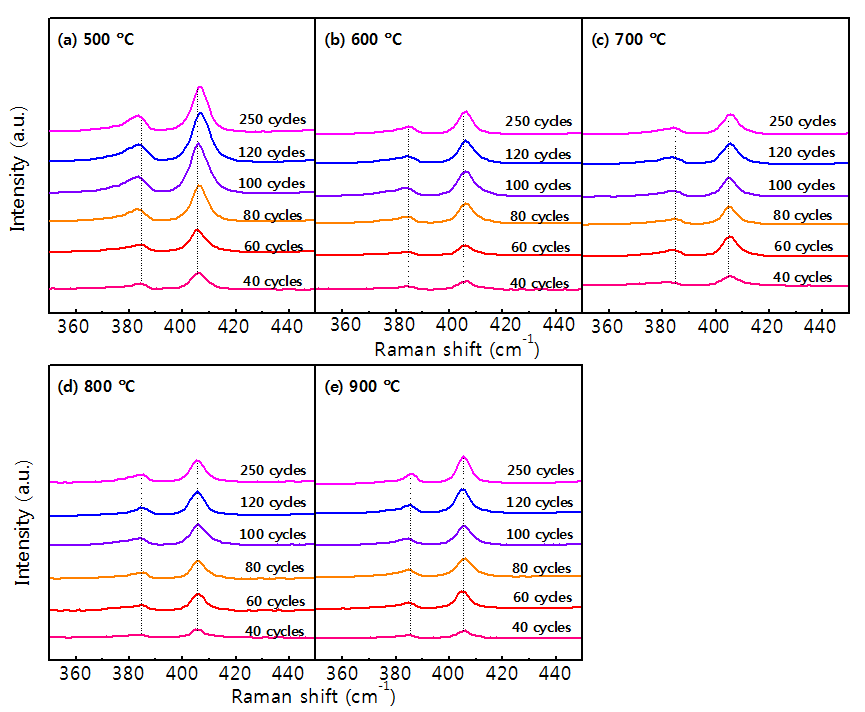


**Figure S3.** Raman spectra of SLS MoS_2_ with an increasing number of process cycles at (a) 500, (b) 600, (c) 700, (d) 800, and (e) 900 °C

**D. Precursor exposure time in the MoS_2_ growth**

Figure S4 shows constant layer number of MoS_2_ nanosheets as the precursor exposing time increased from 2s. Growth saturation was observed after 2s, indicating self-limiting growth characteristics.


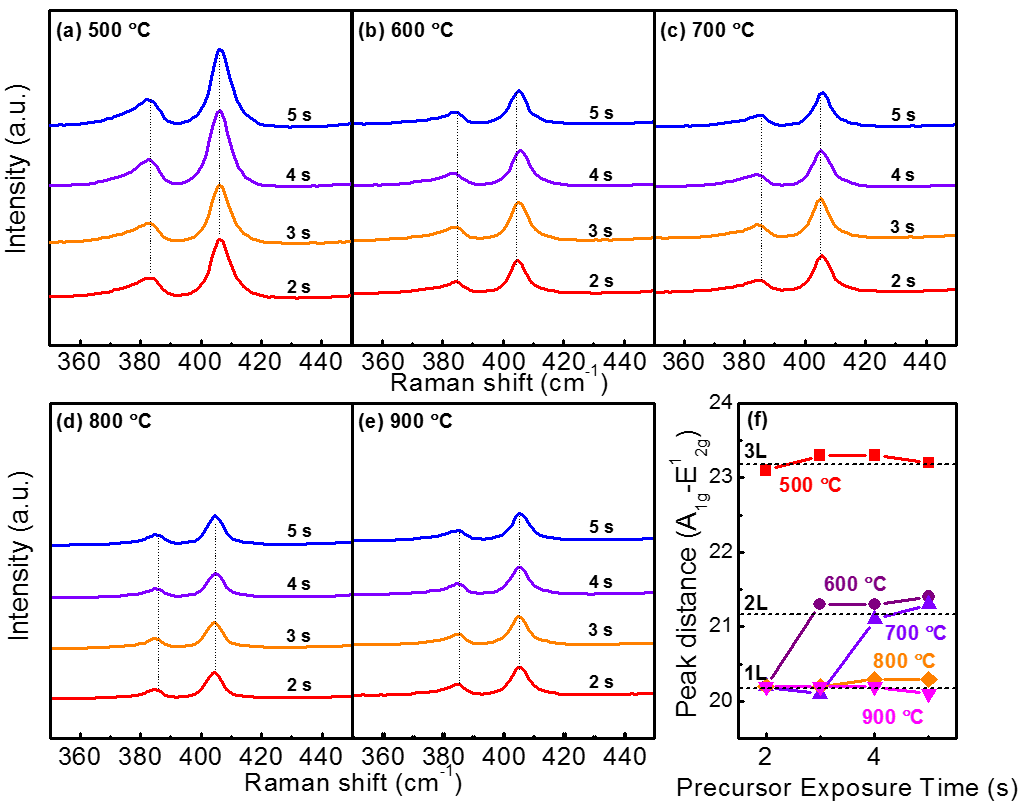


**Figure S4.** Raman spectra of SLS MoS_2_ with increasing precursor exposure time at each growth temperature at (a) 500, (b) 600, (c) 700, (d) 800, (e) 900 ℃, and (f) Raman peak distance for MoS_2_ with precursor exposure time and growth temperatures.

**E. Self-limiting characteristic on sapphire**

Figure S3 shows that with an increasing number of SLS cycles on a sapphire substrate, the maximum number of layers produced is determined by the growth temperature: i.e., two layers at 550 °C, or just one layer at 600 °C.


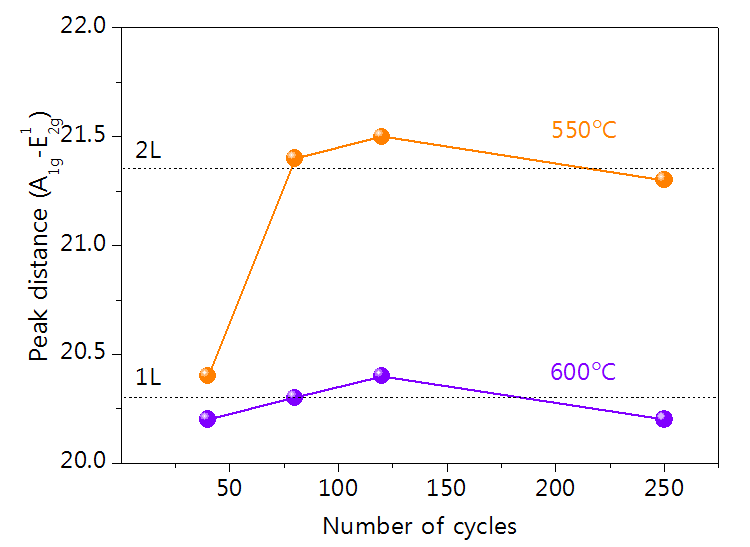


**Figure S5.** Raman peak distances for SLS MoS_2_ formed on a sapphire substrate with an increasing number of SLS cycles at different growth temperatures

**F. Peak distances**


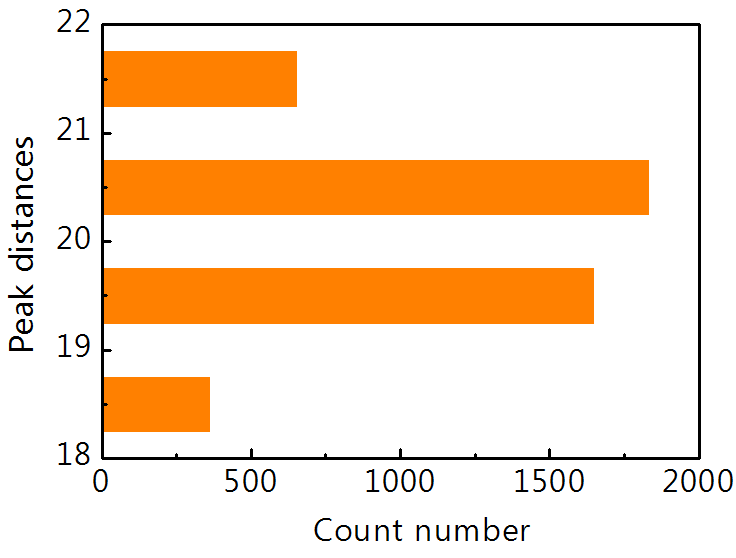


**Figure S6.** Statistical representation of peak distances from the Raman map in Figure 2(a)

**G. HRTEM of 2L and 3L MoS_2_**

The high-resolution transmission electron microscopy (HRTEM) images in Figure S5 show that Moire patterns were observed with both 2L and 3L MoS_2_. The accompanying fast Fourier transform (FFT) images show the layer tilting angles for 2L and 3L MoS_2_.


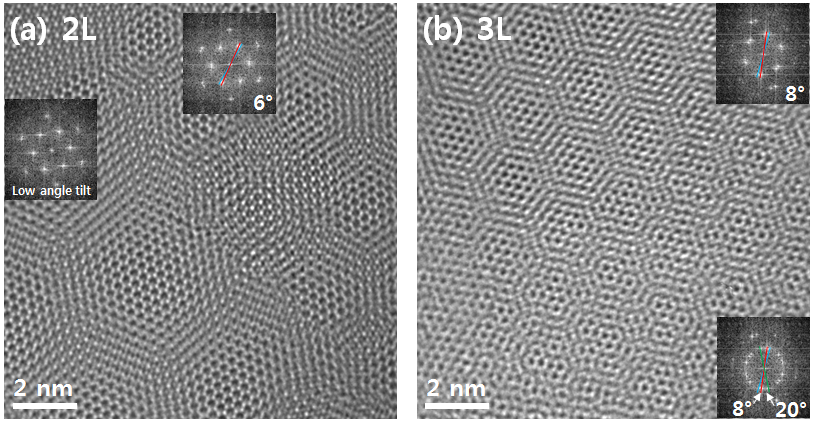


**Figure S7.** HRTEM images and (inset) FFT patterns of (a) 2L and (b) 3L MoS_2_

**H. AFM and Raman measurement of exfoliated WSe_2_**


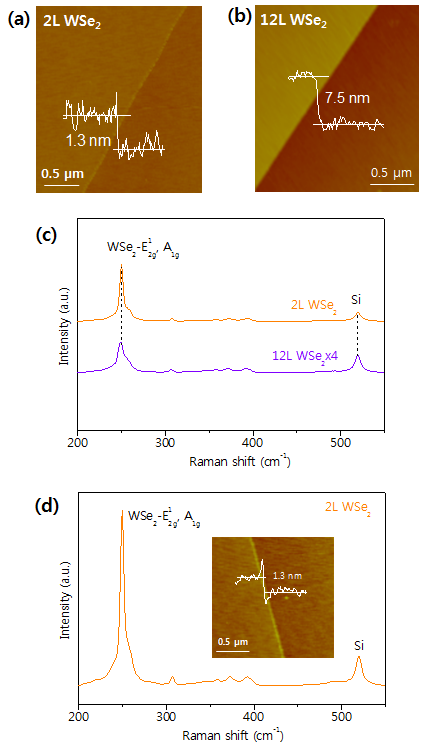


**Figure S8.** AFM images of exfoliated (a) 2L and (b) 12L WSe_2_. (c) Raman spectra of the WSe_2_ flake in Figure 3(a). (d) Raman and (inset) AFM results for the 2L WSe_2_ in Figure 3(d) (as indicated by the white arrow)

**I. Raman and AFM measurement of exfoliated 2L WSe_2_.**

The selective growth of MoS_2_ depending on the thickness of WSe_2_ is explained by the same reduction in surface potential with an increasing number of layers that is seen in the self-limiting mechanism of SLS MoS_2_. This is shown schematically in Figure S7, in which MoCl_5_ molecules chemically adsorb to dangling bonds of the SiO_2_ substrate and grow to form 1L MoS_2_. Similarly, with a 2L WSe_2_ substrate, the positive charge generated between WSe_2_ and the SiO_2_ substrate physically adsorbs MoCl_5_, which subsequently reacts with H_2_S to form MoS_2_. An increasing number of layers, however, creates a screening effect that reduces the influence of the electric field on the WSe_2_, thereby reducing the adsorption energy of the MoCl_5_ molecules and allowing them to more easily desorb from the WSe_2_.


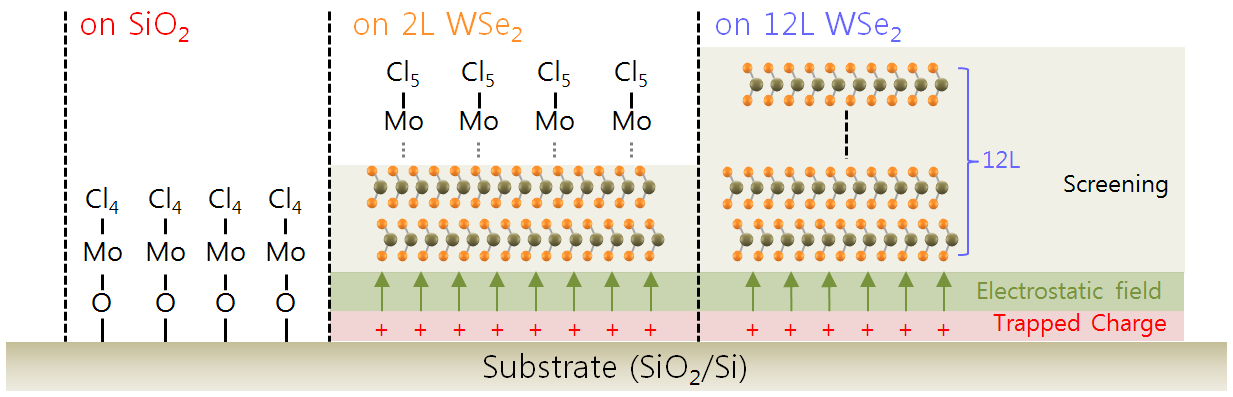


**Figure S9.** Schematic showing the absorption of MoCl_5_ molecules onto a SiO_2_ substrate and WSe_2_ flakes with different layer thicknesses

**J. Gate-tunable characteristic of a MoS_2_/WSe_2_ PN diode**

The gate-tunable characteristic of PN diode could be explained by a variation in carrier density with electrical doping, as in the p-n rectifying region, the majority of carriers in MoS_2_ (electron) and WSe_2_ (hole) are depleted under a reverse bias (Figure S8(a)). At a forward bias, the majority carriers are recombined at an interlayer (Path 1 in Figure S8(b)) that can be described by Shotckely-Read-Hall and Langevin recombination , or the carriers are able to overcome conduction and the valence band offset (Path 2 in Figure S8(c)) , thereby ensuring a continuous current is maintained. With increasing gate voltage, p-type WSe_2_ is electrically doped to n-type and a continuous current is maintained regardless of the bias direction .


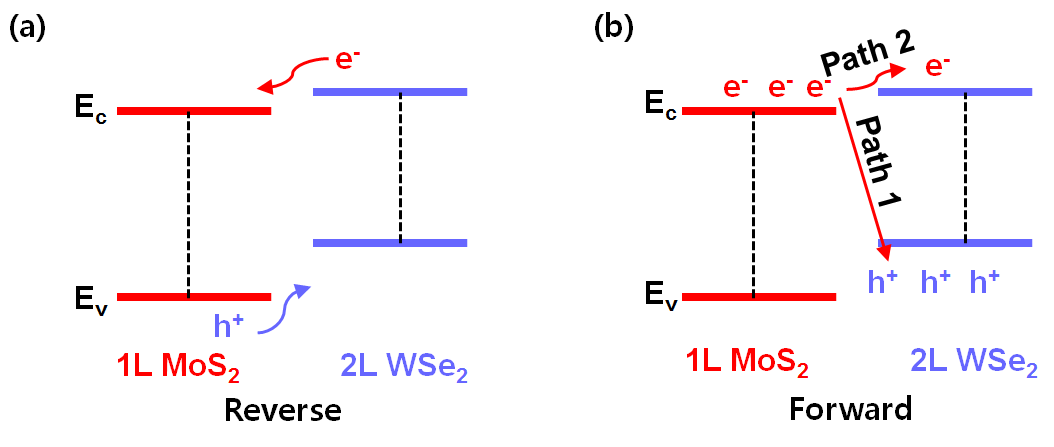


**Figure S10.** Operation of PN diode under (a) reverse and (b) forward bias conditions.

REFERENCES

1. Lee, C.-H., et al., *Atomically thin p–n junctions with van der Waals heterointerfaces.* Nat Nano, 2014. **9**(9): p. 676-681.

2. Roy, T., et al., *Dual-Gated MoS2/WSe2 van der Waals Tunnel Diodes and Transistors.* ACS nano, 2015.

3. Furchi, M.M., et al., *Photovoltaic effect in an electrically tunable van der Waals heterojunction.* Nano letters, 2014. **14**(8): p. 4785-4791.

4. Jariwala, D., et al., *Gate-tunable carbon nanotube–MoS2 heterojunction pn diode.* Proceedings of the National Academy of Sciences, 2013. **110**(45): p. 18076-18080.
